# Serum Colorectal Cancer Biomarkers Unraveled by NMR Metabolomics: Past, Present, and Future

**DOI:** 10.1021/acs.analchem.1c04360

**Published:** 2021-11-22

**Authors:** Ana M. Salmerón, Ana I. Tristán, Ana C. Abreu, Ignacio Fernández

**Affiliations:** Department of Chemistry and Physics, Research Centre CIAIMBITAL, University of Almería, Ctra. Sacramento, s/n, 04120 Almería, Spain

Colorectal cancer (CRC) is a malignant growth, known as polyp, located on the inner lining of the large intestine, which is constituted by the colon and rectum. Its development begins when cells start growing continuously, modifying their shape, size, and other related characteristics.^1^ This can lead to cancer over time, and it can be presented in two ways, as adenomatous polyps (adenomas), which are able to turn into cancer, and as inflammatory or hyperplastic polyps, which are more frequent and in general are not precancerous.^2^

In 2020, colorectal cancer represented the cancer with the third highest incidence worldwide, after breast and lung cancer, estimating more than 1.9 million new cases, and ranked in second place in terms of mortality with a total of 935 173 deaths according to the Global Cancer Observatory GLOBOCAN 2020.^3−5^ The most important predictor of CRC survival is its stage at diagnosis: if an early diagnosis of colon cancer can be provided, a relative 5-year survival rate has been proven to be around 90% for patients diagnosed with localized-stage disease, declining to approximately 71% and 14% for those diagnosed with regional and distant stages, respectively.^1^ Therefore, preventive CRC screening tests are generally recommended to be carried out in the population over 50 years old due to a higher risk of enduring this disease over this age.

Currently, other causes that can lead to the development of these tumors are related to unhealthy lifestyle habits such as being overweight or obese, smoking, intaking processed meat, having a sedentary lifestyle, and excessively consuming alcohol. In addition to these factors, some others that do not depend on the individual can be classified into the following categories: (1) having a predisposition to diseases and conditions such as the presence of polyps in the colon and/or rectum and inflammatory diseases such as Crohn’s disease and ulcerative colitis, (2) having previously suffered from colorectal cancer, which increases the risk of subsequent cancer, and (3) having genetic factors, such as Lynch syndrome and familial adenomatous polyposis (FAP), or family factors, since the incidence has been shown to be higher in those with relatives who have developed colorectal cancer.^6^

Nowadays, the detection of cancerous polyps is carried out by visual analysis of the structure of the colon and rectum with colonoscopy and sigmoidoscopy being the first and the most widely used techniques with the highest sensitivity and detection rate of this kind of pathology. However, colonoscopy procedures have the clear disadvantage of being highly invasive, a fact that implies an increase of certain risks, such as intraperitoneal or extraperitoneal perforation of the colon, along with the requirement of a tedious and not pleasant preparation process prior to the procedure as well as possible anxiety effects.^7−9^ For this reason, noninvasive techniques included in the “omics” sciences, such as genomics, proteomics, transcriptomics, or metabolomics, are in full swing in the field of biotechnology, contributing in a fundamental way to the understanding and prediction of basic biological issues such as cancer diseases.^10,11^ Multiple omics approaches, either alone or in combination, can be applied to explore the heterogeneity of a certain disease and even in a patient’s response to treatment.

Clinical metabolomics aims to identify small molecule metabolites present in patient-derived samples and has attracted much attention to support the discovery of novel biomarkers, which can assess not only the choice of the best treatment for each patient but also the ideal personalized dose regimen. Clinical biomarkers can be dynamic and static. The first ones are commonly employed in patient care and for treatment assessment since they help to define disease progression and the patient's response to the treatment. Static biomarkers are prognostic and aim to predict the clinical response and typically reflect aspects of the physiological state of a patient related to drug treatment response or disease progression dynamics.^12^ Overall, metabolomics is involved in a range of clinical applications, including the identification of diagnostic biomarkers of certain diseases, the elucidation of illness mechanisms, the discovery of novel drug targets, and the prediction of treatment reactions. Along with the response of patients to therapies, it is relevant to be able to create more personalized options, contributing to precision medicine. Thus, pharmacometabolomic studies are showing promising results for predicting drug efficacy and toxicity.^13^ Metabolomics supports the relevance of viewing each individual as a different combination of biochemical, physiological, and environmental interactions.^14^

We perform herein a review of the published investigations dedicated to the research of the metabolic changes produced in blood serum samples of patients with colorectal cancer, mainly involving nuclear magnetic resonance (NMR) alone and/or in combination with other analytical platforms, with the aim of providing a cutting-edge list of potential biomarkers of this disease. Due to the scarce contributions in the most recent years that dealt with the main topic of this Review, the covered time period consisted of 12 years from July 2009 up to June 2021. This Review is also intended to adequately summarize some of the advances made in the subject and to provide a generic application guide for future studies. Some important considerations on how to perform a NMR-based metabolomics project in a clinical setting are also given. Moreover, a brief section mentioning additional NMR-based metabolomics studies on other biological matrices different from serum, such as urine or feces, were included in order to demonstrate the potential and versatility of this technique.

## Metabolomics Undergoing NMR in Clinical Studies

Omic sciences attempt to comprehensively study and interpret the complex interactions between molecules in biological systems. As technological advances progress, omic sciences are becoming more notable in the clinical setting,^15^ allowing the development of earlier personalized diagnoses to patients and in some instances preventing the progress of the disease.^16^

Metabolomics allows one to obtain a picture of the final state of an organism, offering current information on cellular activity.^17^ Until recently, metabolomics has been applied less broadly than other omics, and it has sometimes been referred to as a “complementary analysis” to the rest of them.^18^ This is the case for many reasons: high subject-to-subject and intrasubject variability, a limited number of annotated metabolites, dependence on the use of complex and usually expensive analytical platforms, and the fact that interpretation of the results requires a special combination of technical, statistical, and biological or physiological knowledge.^12,19,20^ Although having controversial factors, this omic science has emerged in recent years as a powerful tool in the search for potential biomarkers associated with diseases,^21^ such as colorectal cancer,^22,23^ and as a source of classification and/or prediction models. Some of the main advantages of this discipline are listed herein: (1) it allows to obtain a significantly smaller data set in comparison to other omics, simplifying data processing; (2) the obtained data (profile of metabolites) is able to faithfully reflect multiple aspects of cellular physiology and the current status of the organisms; (3) the identification and quantification of these metabolites are reliable and reproducible and allow one to correlate the fluctuations on their concentration levels and metabolic fluxes with phenotype information.^24,25^

Metabolomics studies are supported by different high-resolution analytical platforms, such as mass spectrometry (MS) hyphenated to separation methods such as gas (GC) or liquid (LC) chromatography and of course NMR,^26^ enabling to reach the set of metabolites involved in several cellular processes in a certain biological system. NMR is one of the most widely used techniques and is presented as a robust and versatile platform that performs the measurement, identification, and quantification of a large number of metabolites, even in complex mixtures, in a reliable and repetitive way. It simultaneously provides quantitative and, when needed, structural information. In recent years, it has achieved a drastic gain in sensitivity (signal-to-noise ratio) thanks to the use of cryogenically cooled NMR probes, the so-called cryoprobes. NMR has been demonstrated to overcome many of the disadvantages of other analytical techniques, for instance: (1) it performs a nondestructive and noninvasive analysis of the sample, (2) it does not require a previous separation or derivatization step, (3) it does not depend on the ionization of the analytes, (4) there is no need to use a mass analyzer since there is no dependence on the mass-to-charge ratio (*m*/*z*),^27,28^ (5) it has no matrix effect, and (6) the quantification of the metabolites does not rely on calibration curves to quantify the concentration and recovery because only one internal standard is usually added for quantification purposes.^29−31^

Routine “omic” NMR spectroscopy suffers from several drawbacks, but probably, the most important one is the fact that ^1^H NMR complex spectra may be inevitably crowded, which hampers the identification and quantification of metabolites.^32^ Nevertheless, there are strategies that overcome signal overlap, which can be obtained by spreading the resonances in a second dimension using 2D NMR spectroscopy or by applying specific filters, such as Carr-Purcell-Meiboon-Gill (CPMG) or diffusion modules.^33,34^ This and other important considerations will be briefly detailed in the next section.

## Setting a NMR-Based Clinical Metabolomics Study

NMR metabolomics has fully expanded the understanding of cellular and physiological metabolism, helping researchers to identify multiple unexpected biochemical associations in different conditions and diseases, for example, in cancer,^35−37^ autism,^38,39^ infertility,^40,41^ major depression disorder,^42,43^ anorexia nervosa,^44,45^ etc. Since metabolite levels within an individual vary over time, in order to obtain the most satisfactory results and to detect associations with a disease, clinical metabolomics studies must attend some specific considerations for controlling and decreasing within-individual and technical variability through an adequate study design.

Commonly, NMR-based metabolomics studies employ well-known biofluids collected from the patients with the most common being blood serum or urine, where the location of the main peaks and the compositions are already almost established. Some other fluids include cerebrospinal fluid, bile, eye humor, and saliva. Also, tissue extracts are being extensively studied although they eventually give worse results than those from intact tissues directly based on the fact that metabolic changes are usually more concentrated in the tissue itself.^30,46^

In a metabolomics study, the experiment design and sampling method are of utmost importance and must be accurately defined. There are some patient-specific factors that may affect interindividual variation, such as gender, age, weight, or lifestyle, so appropriate selection criteria of the individuals must be applied.^25^ Furthermore, when the aim of the research is the discovery of biomarkers for case-control situations, it can lead to inappropriate oversimplifications, contributing to the presence of intra- and interindividual variability in metabolic signatures. For this reason, the collection of repeated samples in triplicate is of great relevance.^12^ Other important factors that may be taken into consideration in a metabolomics study involve the sample storage, sampling size, and time.^12^ Regarding the former, an adequate temperature for clinical samples, which is −80 °C, is required, allowing the steadiness of the metabolome at least for 6 months.^47,48^

In this section, we describe some relevant aspects to take into consideration when developing a NMR-based metabolomics project in a clinical setting to allow for the optimization of the process.

### NMR Data Acquisition

When measuring biological samples by NMR, it is frequently necessary to perform the suppression of water, especially in body fluids, since they usually present a substantial difference in water concentration regarding their own metabolites. Therefore, experiments such as the presaturation of the solvent signal employing a continuous wave pulse are generally performed. For instance, 1D-NOESY PRESAT, in combination with the presaturation module, introduces a 90° triple pulse sequence that effectively eliminates such signals without causing increased distortions in adjacent signals.^49^ Also, other methods such as excitation sculpting or WATERGATE solvent suppression are occasionally employed.^50^

Furthermore, it may also become necessary to ensure the elimination of signals based on molecular weight for which the so-called diffusion filters are used.^49^ Those employ a combination of radiofrequency pulses and magnetic field gradients, whether monopolar or bipolar, that manages to attenuate signals of smaller molecules, usually those from the solvent employed, although they evidence a disadvantage: the rest of the metabolites also suffer attenuation in their signals to a greater or lesser extent depending on their size. Transverse relaxation time (*T*_2_) filters such as CPMG are also applied, which constitutes the most employed sequence in the study of serum samples^51^ and eliminates signals with a small *T*_2_ that are usually associated with systems with long correlation times generally present in macromolecules such as proteins.^52^

The profiling process, which is in fact, the identification of metabolites in one-dimensional NMR spectra, is carried out through direct assignment using multiplicities and chemical shifts with the help of databases such as the Human Metabolome Database (HMDB), multiple tools available such as the Chenomx software, and some packages available for R such as BATMAN or ASICS. Thus, the confirmation of the assignments is performed using different homonuclear bidimensional spectra such as ^1^H,^1^H–COSY or ^1^H,^1^H-TOCSY and heteronuclear spectra such as ^1^H,^13^C-HMQC, ^1^H,^13^C-HSQC, ^1^H,^13^C-HMBC, ^1^H,^15^N-HMQC/HMBC, and ^1^H,^31^P-HMQC/HMBC, where even more detailed information on the structure of the metabolites is obtained.^53,54^

### Metabolomics Analysis Strategy

Similarly to the rest of the omic sciences, metabolomics studies require large numbers of samples and generate a large amount of data, so reducing their size is a special need in order to obtain a more adequate and correct interpretation of the results. For this purpose, chemometrics methods are employed.^55^ Chemometrics is the discipline that combines mathematical and statistical procedures to extract the most relevant information from the experimental data set, thus improving the process of interpretation and providing quality results.^56,57^ Nowadays, chemometrics techniques are primarily used in chemistry for signal processing, experimental designs, variable reduction, data exploration, multivariate data analysis, and pattern recognition.^58,59^

Metabolomics analyses, in global terms, can be divided according to whether there is some type of prior knowledge about the metabolites of interest or whether there is no information about them.^60^ The first one, targeted metabolomics, focuses on the monitoring of previously selected compounds based on known metabolic pathways or pays attention to those biomarkers strongly associated with the study condition. Thus, these metabolites must be appropriately assigned and quantified in the samples. The second one drives untargeted metabolomics analyses and therefore focuses on the unbiased study of the spectral profile as a whole, considering every single signal present in the sample.^61^ To do this, two basic approaches can be utilized: (a) the fingerprinting and/or (b) the profiling method.^62,63^ The former performs a rapid evaluation of the total metabolites present in the spectra by transforming them into data matrices using the bucketing method (or binning), where small spectral regions or “buckets” with a width between 0.02 and 0.04 ppm are taken and are later used to carry out the pertinent statistical analyses and perform classifications.^64^ The latter consists of studying the entire spectrum using specific peak alignment algorithms and is used to determine the concentrations of all quantifiable metabolites in biological samples, providing useful information from a biochemical point of view.^65^

**Figure 1:**
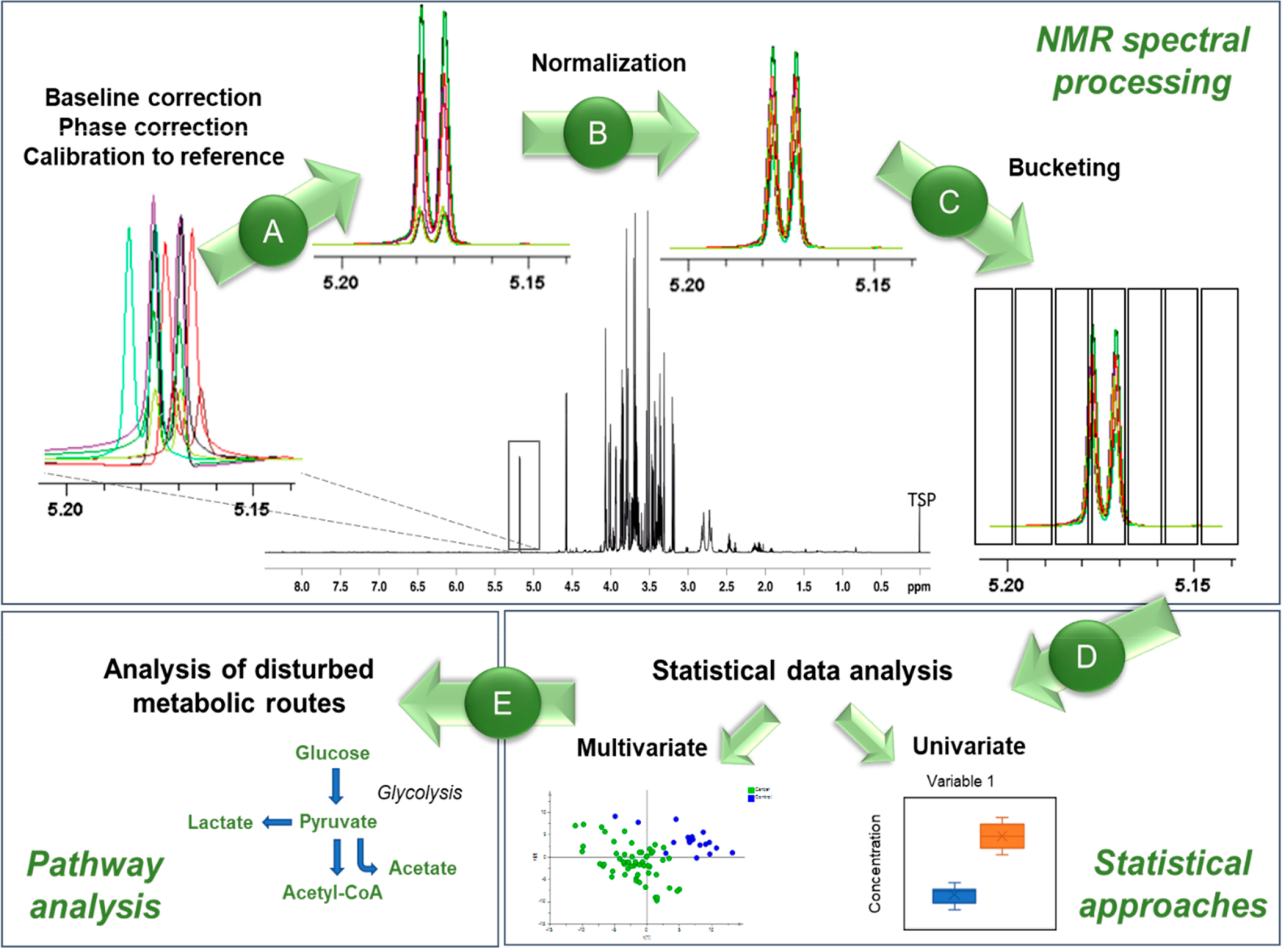
Basic steps of a NMR-based metabolomics study through the fingerprinting method: (A) baseline correction, phase correction, and calibration to the reference, which is usually placed at 0 ppm, (B) NMR spectra normalization to total intensity, (C) bucketing of the spectral data, obtaining a data matrix, which can be subjected to scaling and centering, (D) statistical data analysis, distinguishing between multivariate and univariate approaches, and (E) the analysis of disturbed metabolic routes.

**Figure 2:**
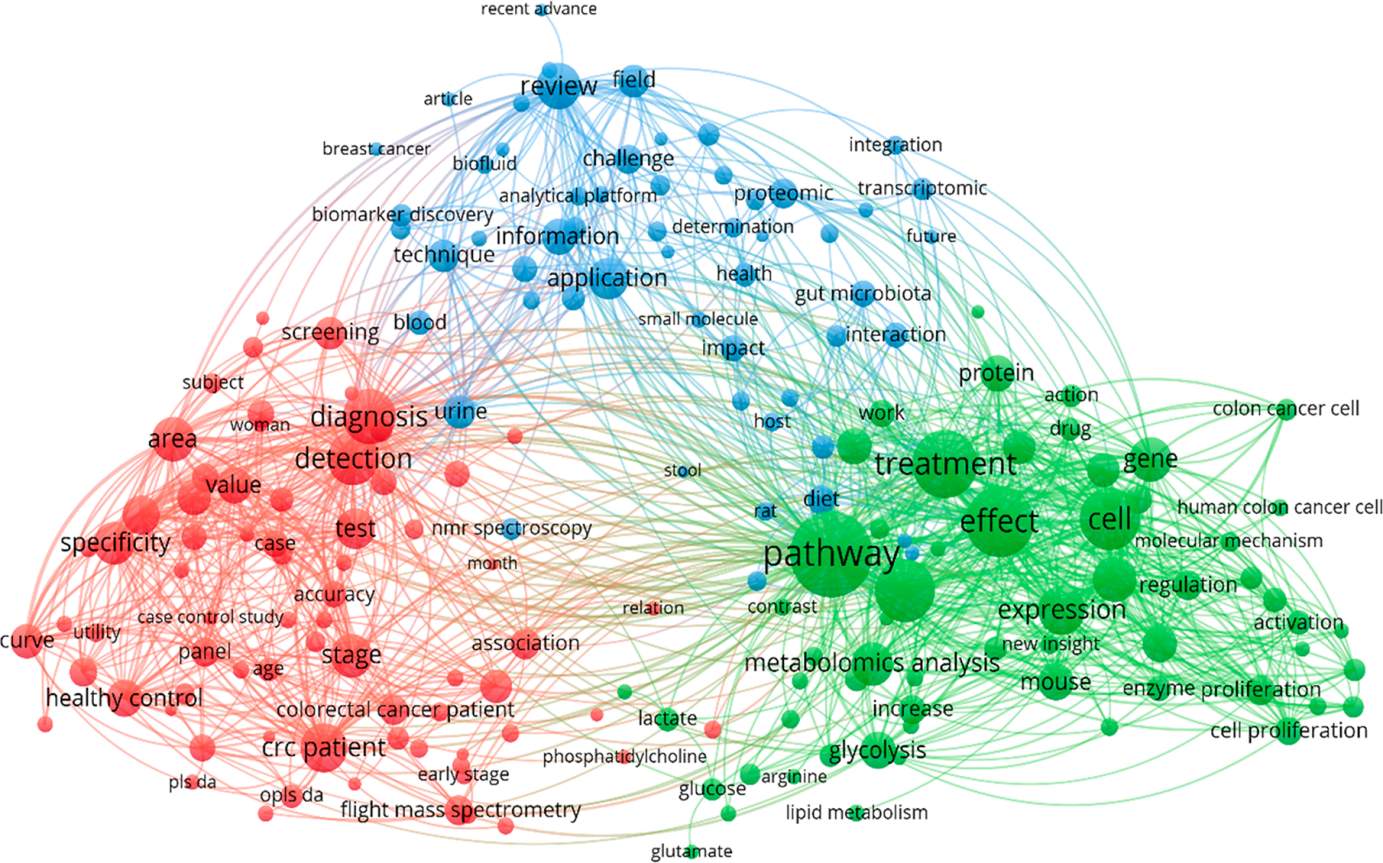
Connectivity network visualization obtained by using the program VOSviewer selecting the *co-occurrence* option. The data set was generated from Web of Science by introducing the keywords “metabolomics” or “metabonomics” and “colorectal cancer” or “colon cancer” or “colorectum cancer”.

**Figure 3:**
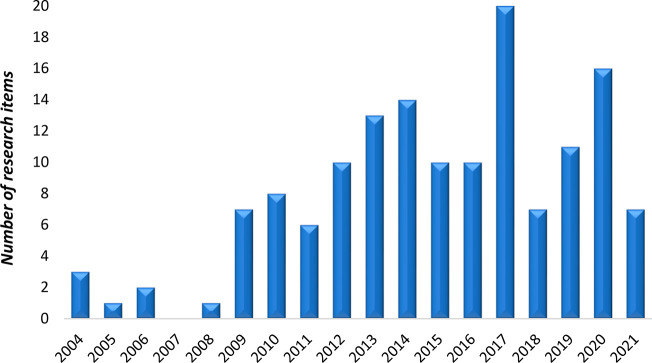
Annual number of publications from 2004 to October 2021. The data set was generated from ISI Web of Science by introducing the keywords “metabolomics” or “metabonomics” and “colorectal cancer” or “colon cancer” or “colorectum cancer” and “NMR” or “nuclear magnetic resonance”.

The process of analysis through the fingerprinting method follows a series of steps, which will be collected and briefly discussed in the following sections: (1) NMR Spectral Processing (Figure 1A–C), (2) Statistical Data Approaches (Figure 1D), and (3) Pathway Analysis (Figure 1E).^66^

### NMR Spectral Processing

#### Baseline Correction, Phase Correction, and Calibration to Reference

This step involves the transformation of the spectral data into their optimal version for the subsequent statistical analysis. It includes the following: checking for the absence of data; adjusting the baseline; referencing the spectrum so that the signal from the internal standard is located at the same chemical shift in all spectra; multiplying the spectrum by functions that soften or accentuate the spectral resolution; applying algorithms that minimize fluctuation in the chemical shifts as a consequence of variations in temperature; suppressing defective spectral regions or areas where there are signal shifts, usually coming from acidic groups or exchangeable protons.^30,64^

#### Normalization

In NMR analysis, identical sample volumes are usually acquired to make all samples comparable with each other. However, in the case of samples corresponding to biofluids, there are multiple external variables that can affect the concentration of metabolites, such as the hydration status of each individual or even possible experimental inaccuracies or technical errors.^53,67^ In order to obtain comparable volumes and concentrations, a normalization step should be applied, which manages the correction of these dilution or concentration factors between samples. In metabolomics, a series of methods are used although normalization is generally achieved by considering the intensity of the total area of the spectrum^68^ and by dividing the values of the peak integration of the buckets by the sum of all of them, so that the sum of all these divisions must provide a value equal to unity.^30,67,69,70^

#### Bucketing

As mentioned before, the total of the metabolites in the NMR spectra are assessed through their transformation into data matrices constituted by minor spectral areas (between 0.02 and 0.04 ppm of the width) called “buckets”. Once the “buckets” table has been obtained, the multivariate statistical analysis of the data is carried out. For this, the purpose of the study must be kept in mind, which may be (a) the visualization of the general differences between samples, such as trends or correlations, (b) the detection of statistical significant differences between groups, (c) the highlight of spectral regions that contribute the most to the observed differences, and/or (d) the construction of a predictive model for the correct classification of new samples.^64^

After this step of bucketing, multivariate analysis techniques are usually used to extract information from the data with the aim of providing biological knowledge on the studied matter.^71^ This data analysis focus on the spectral profile and any information on biological variation can overlap, so centering through the mean of the data is a fairly common step, since it enables to compensate for this problem, focusing on biological variation and the possible differences and similarities between the samples. However, those metabolites that are more abundant in the samples will show higher values in the data table, so they will end up contributing more to the model that is generated later.^69^ In order to avoid this bias, scaling methods are employed. Among the different alternatives available, the most used are (1) unit variance, which compares all metabolites in order to their correlations, increasing its measure error; (2) pareto scaling, which decreases the relative importance of higher values, leaving the data structure relatively complete; (3) range scaling, which compares all metabolites in order to their biological response range; (4) vast scaling, which focuses on metabolites with small fluctuations; (5) level scaling, which focuses on the relative response.^69,70,72^

### Statistical Data Approaches

The obtained “buckets” table must be subsequently subjected to statistical data analysis (Figure 1D) in order to obtain prospective information. For this purpose, statistical methods such as multivariate or univariate analysis can be implemented in metabolomics investigations, offering both of them advantages and disadvantages. Multivariate statistical methods are essential to be incorporated into metabolomics research, since these are able to correlate effects with patterns of metabolites.^73^ These can explain classifications attributable to variations in biological measurements and can create combinations of variables, called components, by their correlations and inter-relationships.^74^

Multivariate analysis techniques are generally divided into (a) unsupervised methods and (b) supervised methods. Unsupervised methods are used to summarize, explore, and discover natural groupings (clusters) of unlabeled data.^75^ Some examples include principal component analysis (PCA), k-means (KM), and partition around medoids (PAM).^64^ In contrast, in supervised methods, a labeled set of training data is employed to estimate or map the input data to the desired output, resulting in a classification problem and allowing the prediction of new (unlabeled) cases.^76−78^ Examples of supervised methods include partial least squares discriminant analysis (PLS-DA), orthogonal partial least squares discriminant analysis (OPLS-DA), k-nearest neighbors (kNN), and artificial neural network (ANN) techniques.^79,80^

Once applied, both unsupervised and supervised methods must be correctly validated to avoid overfitting issues through techniques such as cross-validation or bootstrapping. In addition, there are methods such as the receiver operating characteristic (ROC) curves, where the proportion of false positives generated in the model is controlled via the area under the curve (AUC).^81^ An AUC is a measure of the accuracy of the diagnostic test in which a value of 1.0 indicates a perfect test, whereas an AUC value of 0.5 shows the test is no better than random chance, and therefore, it has no diagnostic or prognostic value. It is important to mention that special precaution must be taken to interpret AUCs obtained from a small number of samples since they are inherently noisy.^82^

Occasionally, multivariate analysis techniques can ignore important variables, as all metabolites are concurrently studied. For this reason, univariate analysis is a critical phase in metabolomics research, which can also assist in the determination of those metabolites with the strongest response under the investigated conditions. However, it is important to highlight that this kind of analysis does not consider inter-relationships between metabolites concentrations.^74^ In order to find statistical significance in sample comparisons, methods such as the Student’s *t*-tests or the Wilcoxon test are commonly applied when comparing two groups, while the analysis of the variance (ANOVA) or Kruskal–Wallis tests are utilized when having more than two assemblies.^83^

In order to evaluate the possible misconceptions related to *p* values and confidence intervals, Bonferroni and Bonferroni-Holm and Sidak corrections can be applied to mitigate Type I errors (related to the improper rejection of the null hypothesis, such as a false positive),^84^ contributing to the control of the general error proportion. Thus, the Benjamini-Hochberg approach can be employed for the assessment of false discovery rates in univariate analysis.^64,74,85^

### Pathway Analysis

The final objective of the metabolomics studies, illustrated in Figure 1E, is the correct interpretation of the results obtained in the statistical analysis by recognizing the up- and downregulated biomarkers and the disturbed metabolic pathways that may be affected by the condition/disease under study. There are multiple databases, such as KEGG, Reactome, and MetaCyc, that list different metabolic pathways and their involved metabolites. In addition, there are also online tools that help with the analysis and understanding of the data, such as MetaboAnalyst,^86^ which examines the metabolites present in the biological matrix, providing the possible involved pathways and, therefore, helping in the assessment of the biological importance of the results. In addition, to obtain a complete analysis and knowledge of the subject, it is advisable to consult previous publications that have been able to provide relevant information on the subject in question.^64,86^

## NMR Analysis of Serum Samples Obtained from Patients with Colorectal Cancer

The working data set constituting a total of 687 publications was obtained from a Web of Science search^87^ in the Web of Science Core Collection by introducing the keywords “metabolomics” or “metabonomics” and “colorectal cancer” or “colon cancer” or “colorectum cancer” and covering the period from 2004 to October 2021. Figure 2 shows a network visualization of the most shared keywords provided by the VOSviewer software^88^ when applied to this data set.

As it is observed, some clusters of words were established to emphasize, for example, the relevance of multivariate analysis techniques in the metabolomics research of CRC (through the terms “curve”, “auc”, “roc”, “opls-da”, “pls-da”, “accuracy”, “specificity”, “sensitivity”, “value”, “area”, and “test”), the importance of NMR spectroscopy and mass spectrometry in this topic (with the terms “NMR spectroscopy” and “flight mass spectrometry”), and the description of some colorectal cancer biomarkers and disturbed pathways reported by several metabolomics studies of this disease (such as “glycolysis”, “lactate”, “glutamate”, “glucose”, “arginine”, and “lipid metabolism”).

When a second search is performed by introducing “NMR” or “nuclear magnetic resonance” to the same previous keywords, the resulting output was reduced to a set of 146 contributions published from 2004 to October 2021. The following graphic illustrates a positive trend in which a considerable increase in the number of publications on this topic occurred starting in 2009. As previously discussed, analytical techniques have progressed and improved over the last years, especially NMR, which has increased its sensitivity by up to a factor of 5 mostly due to the development of cryoprobes, which have contributed to the development of metabolomics as an increasingly applied field of research, as the upward trend of Figure 3 reflects.

**Figure 4:**
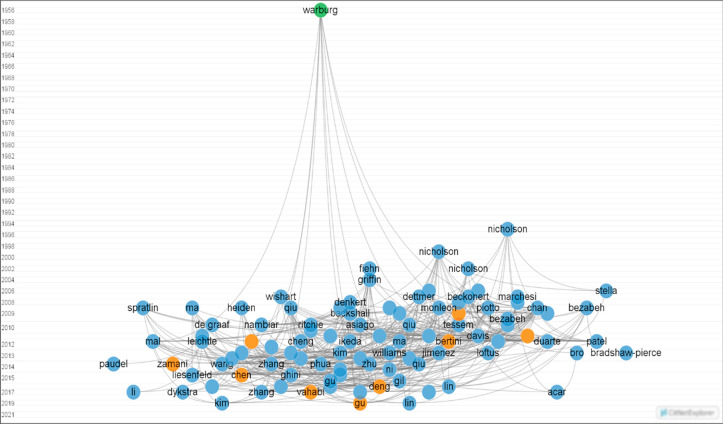
Network of citations obtained by using the program CitNetExplorer. The data set was generated from Web of Science by introducing the keywords “metabolomics” or “metabonomics” and “colorectal cancer” or “colon cancer” or “colorectum cancer” and “NMR” or “nuclear magnetic resonance”.

An analysis of some of these publications was further performed using the CitNetExplorer software^89^ to obtain a citation network including the articles, reviews, and book chapters involved (Figure 4). As it could be observed, all of these publications conjoin in the popular publication of 1956 by Warburg et al. ( marked in green)^90^ in which the Warburg hypothesis, which explains the alleged root cause of cancer, was formulated. The publications that employ serum samples for the NMR-based metabolomics analysis of colorectal cancer, which constitutes the main topic of the current review, appear in orange, while the articles marked in blue are those focused on the analysis of tissue, feces, and urine samples, some of which will be also mentioned further below. A total of 10 contributions were obtained for the former case, including Ludwig et al.,^91^ Backshall et al.,^92^ Bertini et al.,^93^ Farshidfar et al.,^94^ Zamani et al.,^95^ Chen et al.,^96^ Deng et al.,^97^ Vahabi et al.,^98^ Gu et al.,^7^ and Di Donato et al.^99^

Table 1 highlights some of the most important metabolomics investigations dedicated to the study of colorectal cancer specifically in serum samples through NMR with the general aim of obtaining biomarkers of this disease.

**Table 1:** List of Metabolomics Studies of Colorectal Cancer Covered in This Manuscript until June 2021 in Chronological Order^a^

reference	technique	NMR probehead	NMR sample size	sample volume	multivariate analysis	aim	conclusions^c^
Ludwig et al.^91^ (2009)	^1^H NMR (800 MHz)	HCN cryogenic probe	38 CRC,^b^ 8 adenoma, 19 controls	-	PCA, PLS-DA	identification of CRC and adenoma-positive biomarkers	higher levels of Lac, Pyr, Acet, Aco, and 3-HB in CRC samples
Backshall et al.^92^ (2011)	^1^H NMR (600 MHz)	5 mm triple resonance TXI probe	52 patients	200 μL	PLS-DA	prediction of toxic effects on patients undergoing treatment with capecitabine	differences in the serum metabolic profile between pretreatment and subsequent toxicity; the lipid profile provides information on the risk and possible severity of toxicity
Bertini et al.^93^ (2012)	^1^H NMR (600 MHz)	CPTPI probe	153 metastatic CRC, 139 controls	300 μL	PLS-DA, SVM, CA	identification of metastatic CRC biomarkers and predict overall patient survival	lower serum levels in CRC samples of Ala, Cit, Leu, Pyr, Tyr, and Val and higher content of 3-HB, Acet, Form, Glo, lipids, GlyP, Phe, and Pro; this could lead to a signature to predict overall survival
Farshidfar et al.^94^ (2012)	GC-MS, ^1^H NMR (600 MHz)	5 mm triple resonance TXI probe	42 coloregional CRC, 45 liver-only CRC, 25 extrahepatic metastases	-	PCA, OPLS-DA	identification of occult metastases biomarkers in CRC samples	serum metabolic profile changes with metastases and with sites of disease
Zamani et al.^95^ (2014)	^1^H NMR (500 MHz)	-	33 CRC, 33 controls	600 μL	PCA, PLS-DA	identification of CRC biomarkers	decrease in the levels of Pyx, Oro, AdeH, Pya, Glyc, β-Leu, MetCy, Tau, 3-HB, AcChol, 3-HV, Fuc, Chol, and PCar and increase in Gly in CRC samples; the LDA/DCA ratio is considered a possible CRC biomarker
Chen et al.^96^ (2015)	^1^H NMR (500 MHz)	HCN cryogenic probe	44 polyps, 58 controls	530 μL	seemingly unrelated regression	identification of polyps-associated biomarkers	eleven groups of metabolites showed alterations between patients with polyps and healthy controls
Deng et al.^97^ (2016)	LC-MS, ^1^H NMR (500 MHz)	5 mm triple resonance TXI probe	28 CRC, 44 polyps, 55 controls	530 μL	PLS-DA	identification of CRC- and polyps-associated biomarkers	higher levels of Glc, lower levels of Ade, and alterations in Pyr and Gln levels in CRC samples; decrease in Ort and increase in Ade in polyp samples
							alterations in Fum, Cit, Oxa, Lin, and lipids in both groups
Vahabi et al.^98^ (2017)	^1^H NMR (500 MHz)	-	8 CRC stage 0-I, 8 CRC stage I–IV	600 μL	OPLS-DA	identification of CRC biomarkers in different disease stages (0 to I and II to VI)	lower levels of Pyx, increase in Gly, Cholt, Tau, CholEs, and Den in the CRC group (II to IV stages)
Gu et al.^7^ (2019)	^1^H NMR (600 MHz)	-	40 CRC, 32 polyps, 39 controls	300 μL	PCA, PLS-DA, OPLS-DA, RF, SVM	identification of CRC and polyps-associated biomarkers	increase in the levels of Lac, Gly, Ser, Chol, and 3-HB and decrease of Cit and Suc in CRC samples; higher levels of Lac, Glut, Chol, PUFA, and NaGly and lower levels of Acet, Glo, Gln, Ala, and Asp in polyps samples
							Acet/Glo and Lac/Cit ratios considered as possible polyps and CRC biomarkers, respectively
Di Donato et al.^99^ (2021)	^1^H NMR (600 MHz)	-	65 CRC relapse free, 29 relapsed, 75 metastatic CRC	1 mL	PCA-CA-kNN	prediction of prognosis and identification of metastatic CRC biomarkers	lower levels of Gln and His in the metastatic CRC group

a Symbol: “-” denotes studies where the specific information was not provided.

b Colorectal cancer.

c In samples corresponding to colorectal cancer, abbreviations are as follows. Acet: acetate; Aco: acetoacetate; AcChol: 7-acetocholesterol; Ade: adenosine; AdeH: s-adenosylhomocysteine; Ala: alanine; Asp: aspartate; Cholt: cholesterol; CholEs: cholesteryl ester; Chol: choline; Cit: citrate; DCA: deoxycholic acid; Den: deoxyinosine; For: formate; Fuc: 3-fucose; Fum: fumarate; Glc: glucose; Glut: glutamate; Gln: glutamine; Glo: glycerol; Gly: glycine; Glyc: glycocholic acid; GlyP: glycoproteins; His: histidine; 3-HB: 3-hydroxybutyrate; 3-HV: 3-hydroxyisovaleric acid; Lac: lactate; Leu: leucine; Lin: linolenic acid; LDA: lithocholic acid; MetCy: 5-methylcytidine; NaGly: N-acetyl glycoproteins; Ort: oroate; Oro: orotidine; Oxa: oxaloacetate; PCar: l-palmitoylcarnitine; Phe: phenylalanine; Pro: proline; PUFA: polyunsaturated fatty acids; Pya: pyridoxamine; Pyx: pyridoxine; Pyr: pyruvate; Ser: serine; Suc: succinate; Tau: taurocholic acid; Tyr: tyrosine.

The study by Ludwig et al.^91^ employed multidimensional 2D-^1^H,^1^H-TOCSY NMR spectra for the metabolomics analysis of 38 serum samples positive in colorectal cancer, 8 of them also being confirmed in adenoma, and 19 control samples. The application of PCA and PLS-DA models to NMR data allowed them to conclude that the cancerous samples showed higher levels of lactate, pyruvate, and ketone scaffolds (acetate, acetoacetate, and 3-hydroxybutyrate) than the control ones. In this work, they were able to significantly reduce the measurement time of the ^1^H,^1^H-TOCSY spectra due to the substitution of the Fourier transform by the Hadamard transform, thus being able to deconvolute crowded NMR spectra.^91^

In 2011, the team of Keun^92^ accomplished the first study to report the capacity of ^1^H NMR metabonomics to predict adverse effects and toxicity severity associated with the administration of the chemotherapy medication capecitabine in serum samples of patients with colorectal cancer. For this purpose, a generated PLS-DA model was able to correlate the presence of higher levels of polyunsaturated fatty acids and choline phospholipids with higher grade toxicity over the treatment period; however, this model did not reach significance by cross-validation.^92^

Later, Bertini et al.^93^ employed ^1^H NMR to study the metabolic profile of 153 samples of metastatic colorectal cancer serum and 139 control samples with the aim of obtaining valid biomarkers and predicting patient survival. First, they applied PLS-DA for dimension reduction, followed by a canonical analysis (CA) evaluation that revealed good discrimination and a support vector machine (SVM) model for classification. In this analysis, they observed lower and higher levels of 6 (alanine, citrate, leucine, pyruvate, tyrosine, valine) and 8 (3-hydroxybutyrate, acetate, formate, glycerol, lipids, glycoproteins, phenylalanine, and proline) metabolites, respectively, in metastatic colorectal cancer samples, leading to a possible metabolic signature, which may offer an independent tool to predict overall survival. In addition, they verified different metabolic shifts between patients with shorter and longer survivals.^93^

In the same year, Farshidfar et al.^94^ conducted research employing GC-MS and ^1^H NMR with the aim of distinguishing the stage of colorectal cancer in 42 serum samples of patients with coloregional colorectal cancer (cancer stages II and III), 45 samples of patients with liver-only metastases, and 25 samples of patients with extrahepatic metastases (cancer stage IV in both cases). A PCA exploratory analysis followed by an OPLS-DA model allowed them to differentiate serum metabolic profiles of patients with metastases and between metastases appearing in different organs.^94^

In 2014, Zamani et al.^95^ carried out a metabolomics study using ^1^H NMR of 33 serum samples corresponding to a positive group in colorectal cancer and 33 control samples with the aim of obtaining a prediction model and possible biomarkers. The application of PCA and PLS models to ^1^H NMR data showed a positive discrimination between both groups, caused by a decrease in the levels of pyridoxine, orotidine, *s*-adenosylhomocysteine, pyridoxamine, glycocholic acid, β-leucine, 5-methylcytidine, taurocholic acid, 3-hydroxybutyric acid, 7-acetocholesterol, 3-hydroxyisovaleric acid, l-fucose, cholesterol, and l-palmitoylcarnitine for the cancer group together with an increase in glycine. In addition, they highlighted the ratio of lithocholic acid/deoxycholic acid as a possible biomarker of colon cancer.^95^

Furthermore, Chen et al.^96^ conducted an investigation into the ^1^H NMR metabolic profile of 44 patient samples with colon polyps and 58 control samples along with numerous demographic parameters, performing seemingly unrelated regression (SUR) for the correlation of the metabolites and the biological groups. They were able to obtain valine as a slightly significant metabolite for patients with polyps, as they had a reduced sample size, but could report 11 groups of metabolites that were significantly different between polyps and control samples.^96^

A year later, in 2016, the group of Raftery^97^ carried out a metabolomics study using LC-MS and ^1^H NMR on serum samples from a positive group for colorectal cancer of 28 subjects, a total of 44 individuals with polyps, and a third group of 55 controls. They generated an algorithm where all variables were examined, removing one of them in each iteration and employing the remaining ones for PLS-DA. Variables with the highest prediction accuracy for the test samples in Monte Carlo Cross Validation (MCCV) were kept for the subsequent iteration, resulting in a 30% test set and a 70% training set, the portion where PLS-DA was performed to predict the classification of the test set samples. Colon cancer samples displayed higher levels of glucose, lower levels of adenosine, and alterations in the levels of pyruvate and glutamine, while a decrease in orotate and an increase in adenosine were found in the group positive for polyps. Alterations in the levels of amino acids, fumarate, citrate, oxaloacetate, linolenic acid, and lipids were observed for both cancer and polyp groups compared to the controls.^97^

Vahabi et al.^98^ investigated the differences in the ^1^H NMR metabolic profile of 16 colorectal cancer samples between 0–I stages (8 samples) and I–IV stages (8 samples), similarly to Farshidfar et al.^94^ An OPLS-DA model showed decreased levels of pyridoxine and increased contents of glycine, cholesterol, taurocholic acid, cholesteryl, and deoxyinosine for the II–IV stages of colorectal cancer samples.^98^

In 2019, Gu et al.^7^ conducted a ^1^H NMR metabolomics analysis of 40 serum samples from colon cancer patients, 32 samples positive for polyps, and 38 controls, and several models [PCA, PLS-DA, OPLS-DA, random forest (RF), and SVM methods] were applied to the ^1^H NMR data to identify possible biomarkers. A total of 23 metabolites were elucidated, reporting an increase in the levels of lactate, glycine, serine, choline, and 3-hydroxybutyrate and a decrease of citrate and succinate for colorectal cancer samples. Also, higher levels of lactate, glutamate, choline, polyunsaturated fatty acids, and N-acetyl glycoproteins and lower levels of acetate, glycerol, glutamine, alanine, and aspartate were found for the polyps samples. Furthermore, they could determine that the acetate/glycerol and lactate/citrate ratios were important biomarkers for the presence of polyps and colorectal cancer, respectively.

Recently, Di Donato et al.^99^ hypothesized that NMR-based metabolic fingerprinting could improve risk stratification in patients with early colorectal cancer and investigated serum samples of 94 elderly patients with early stage colorectal cancer (65 relapse free and 29 relapsed after a 5 year median followup) and 75 elderly patients with metastatic colorectal cancer. Prognosis was assessed using Kaplan–Meier curves, and a PCA-based kNN analysis was able to distinguish between relapse free and metastatic colorectal cancer groups, mainly due to lower levels of glutamine and histidine in patients with metastatic colorectal cancer.^99^

### Correlation of Main Serum Biomarkers to CRC

In general, it has been proven that the most relevant metabolites found to be important biomarkers associated with colorectal cancer are widely related to carbohydrate metabolism, involving gluconeogenesis^96,93^ and specially glycolysis,^7,91,94,96,97,100,101^ since an increase in activity in this pathway can lead to an increase in malignant tumors, known as the Warburg effect.^90^ This process involves an abnormal accumulation of glucose, pyruvate, and lactate (initial, intermediate, and final metabolites of glycolysis, respectively), as reported in many of the studies previously discussed.^7,97,91,93^ Furthermore, lower amounts of other metabolites related to glycolysis, such as citrate and succinate, were also indicated as part of this Warburg effect.^7,90^ Citrate is also involved in the citric acid cycle in combination with fumarate and oxaloacetate, and its levels were found to change in the serum of individuals with CRC cancer and polyps when compared to the control ones.^7,97^

In turn, a high demand for amino acids by the growing tissues can also cause alterations in the metabolic routes associated with these compounds, and a consequent decrease in their levels in carcinogenic samples has been reported, e.g., arginine, glutamine, proline, alanine, aspartate, and glutamate,^7,95,97,96,98^ accompanied by an accumulation of ketone scaffolds such as acetate, acetoacetate, and 3-hydroxybutyrate.^95,91^ Additionally, Deng et al.,^97^ Gu et al.,^7^ and Zamani et al.^95^ reported a decrease in the levels of unsaturated and polyunsaturated fatty acids, possibly due to perturbations in the metabolisms of glycerolipids and fatty acids. Moreover, the biosynthesis of primary bile acids and the metabolism of vitamin B6 were referenced among others (cyanoamino acid, thymine, methane, glutathione, fucose, and mannose metabolisms) by Zamani et al.^95^ and Vahabi et al.^98^ Alternatively, Farshidfar et al.,^94^ focused on the comparison among serum samples from individuals with coloregional and liver-only metastases, reporting an accelerated galactose metabolism being involved in colorectal cancer samples. Also, changes in the metabolism of purine were commonly observed by Vahabi et al.^98^ and Deng et al.^97^ as well as in choline metabolism by Gu et al.^7^

### Multivariate Data Analysis Methods

Most of the studies reviewed herein applied the unsupervised technique of PCA as a first step for the discrimination between groups and to obtain potential biomarkers of colorectal cancer with the exception of Bertini et al.^93^ who employed for this purpose another technique of this sort, canonical analysis (CA). It is worth mentioning the research developed by Di Donato et al.,^99^ in which a PCA in combination with CA and kNN was applied for the discrimination between groups. On the contrary, Backshall et al.,^92^ Chen et al.,^96^ Deng et al.,^97^ and Vahabi et al.^98^ did not employ PCA in their research. Further, for the application of unsupervised techniques, supervised linear multivariate techniques such as PLS-DA^7,95,97,91,93,92^ and OPLS-DA^7,94,98^ were generally applied, and the associated biomarkers were determined using different methods. Some of these studies^7,97,94^ selected the variables associated with the discrimination between disease and healthy individuals based on their variable importance in projection (VIP) index values given by supervised models, in which those variables with a VIP value greater than 1 were considered statistically significant for the model. Chen et al.^96^ employed other statistical approaches like seemingly unrelated regression for the identification of significant biomarkers.

Some studies also included nonlinear methods for the classification and identification of the most discriminant metabolites, for instance, the study by Bertini et al.,^93^ in which a SVM model was implemented to the PLS scores by applying the nonparametric Kruskal–Wallis rank-sum test for the continuous variables and the Fisher exact test for the categorical ones. Moreover, Gu et al.^7^ implemented an RF classifier in combination with the correlation coefficients of several discriminating metabolites found from a previous OPLS analysis and selected the most important biomarkers according to their frequency of being chosen by the algorithm, and later, a SVM model was applied in order to validate the obtained results. The results were supported by employing the area under the ROC curve.

In order to validate the linear models of PLS-DA and OPLS-DA, several of the studies employ the parametric cross-validation test CV-ANOVA, while the precision of the nonlinear models was evaluated using AUC-ROC curves, which make it possible to verify the proportion of false positives derived from confusion matrices in combination with their respective confidence intervals (CIs). Lastly, most of the studies carried out metabolomics pathway analysis by performing the Holm-Bonferroni correction with the aim of determining the most enriched ones.

### Analytical Platforms, Acquisition Parameters, and Processing

In general, the ^1^H NMR-based metabolomic studies mentioned in this Review demonstrate a great power of prediction, classification, and selection of biomarkers associated with colorectal cancer; however, although the current trend continues to increase, the number of articles that relied solely on ^1^H NMR is still lower. Indeed, some of the studies included in this Review performed the analysis of the metabolic profiles in combination with other analytical techniques. For example, in addition to NMR, Deng et al.^97^ applied LC-MS, while Farshidfar et al.^94^ utilized GC-MS. It is of great importance to highlight these differences since, depending on the equipment used, different metabolites can be detected depending on the sensitivity and specificity of each platform.

Concerning the publications using ^1^H NMR, the data acquisition and processing parameters employed could also be a cause of variability of the results. In general, most of the discussed studies in this Review implemented the sequence CPMG to suppress resonances involving high-molecular weight molecules,^91−93,95−99^ some of them solely or in combination with the presaturation of the water signal, as a method of erasing the water signal from the serum samples.^7,91,92,94,96,97,99^ Regarding the data processing parameters, including the type of normalization, scaling, and/or transformation applied, these studies showed a general lack of consensus among them and generally applied different statistical approaches. In this sense, depending on the scaling method used, several types of signals can be prioritized, which can lead to misleading conclusions. Therefore, except for Bertini et al.,^93^ most articles presented quite a small sample size, an aspect that could have conditioned many of the statistical results achieved, resulting in the possible variation of some of the metabolites envisaged as biomarkers.

Differences in the selection of research participants, targeted population groups, and sampling procedures described in each research study should be also considered. Generally, there is a tendency to study differential metabolites between a group of colorectal cancer, overall involving metastases, and a control group. Nevertheless, some of the studies mentioned herein also focused on the study of metabolic differences between samples from patients with polyps and controls. In contrast, the study by Farshidfar et al.^94^ focused equally on the location of the cancer, making a distinction between coloregional, liver-only, and extrahepatic as well as on the stage of this disease, a factor that the team of Vahabi et al.^98^ also studied. On the other hand, Backshall et al.^92^ employed samples derived from patients before being treated with capecitabine in order to relate their profiles to subsequent treatment toxicity, while Di Donato et al.^99^ distinguished between early colorectal cancer patients with and without relapse and elderly patients with metastatic disease.

## Other Matrices Analyzed through NMR Metabolomics in the Quest of CRC Biomarkers

Apart from serum, there are plenty of studies about colorectal cancer that apply NMR metabolomics in other matrices. Table 2 shows seven of the most relevant studies with the principal aims and results of each one being described. These include three main types of matrices: fecal, tissue, and urine samples.

**Table 2:** List of Metabolomics Studies of Colorectal Cancer in Other Matrices until March 2019 in Chronological Order^a^

reference	technique	matrix	NMR probehead	NMR sample size	sample volume	multivariate analysis	aim	conclusions^c^
Bezabeh et al.^102^ (2009)	^1^H NMR (400 MHz)	fecal extracts	flow probe	111 CRC,^b^ 412 controls	500 μL	LDS (linear discriminant analysis)	noninvasive detection of CRC	identified spectral differences important for the detection of colorectal cancer
Chan et al.^105^ (2009)	^1^H NMR (600 MHz), GC-MS	colon mucosae	HR-MAS probe	31 patients	10 mg	OPLS-DA	description of metabolic signatures that discriminate malignant from normal mucosae	higher levels of Lac, Gly, PC, PE, Urd, fecal bile acids, and Cholt and lower levels of Glc, ArA, Mal, Fum, and lipids in CRC samples
Piotto et al.^106^ (2009)	^1^H NMR (500 MHz)	tumoral and healthy tissues	4 mm double resonance (^1^H, ^13^C) gradient HR-MAS probe	44 patients	15/20 mg	PCA, PLS-DA	characterization of the metabolic fingerprint of tumoral and healthy tissue samples from patients affected by primary colorectal adenocarcinoma	elevated amounts of Tau, Glu, Asp, and Lac in colorectal biopsy tissues and a high amount of MI and β-in healthy tissues
Monleón et al.^103^ (2009)	^1^H NMR (600 MHz)	fecal water extracts (stool samples)	triple-resonance ^1^H/^13^C/^15^N probe	21 CRC, 11 controls	500 μL	PCA	identification of potential diagnostic markers of CRC	a low SCFA (Acet and But) concentration seems to be the most effective marker for CRC.; Pro and Cys also displayed a correlation with the disease
Jiménez et al.^107^ (2013)	^1^H NMR (400 MHz)	tumor and adjacent mucosa	TBI HR-MAS probe	26 patients	10 mg	OPLS-DA	find potential biomarkers of CRC in both matrices	discrimination between T- and N-stages; increased levels of Tau, Isogln, Chol, Lac, Phe, and Tyr, and decrease in lipids and triglycerides for tumor tissues
								discrimination between T- and N-stages and accurate predictive capability of 5-year survival for adjacent mucosa
Wang et al.^108^ (2017)	^1^H NMR (400 MHz)	urine	-	55 CRC, 40 controls	600 μL	PCA, OPLS-DA	find metabolic variations between CRC and healthy controls and differentiate between CRC stages	16 potential biomarkers were identified for CRC; metabolic profiles from early stage CRC and esophageal cancer patients were also distinguishable
Kim et al.^109^ (2019)	^1^H NMR (500 MHz)	urine	-	92 CRC, 156 controls	500 μL	OPLS-DA	explore the potential of urine NMR metabolomics as a diagnostic tool for early detection of CRC, compare patients with CRC neoplasia at various stages and healthy controls	preinvasive CRC neoplasia, advanced CRC, and healthy controls groups were statistically discriminated with high sensitivity and specificity; Tau, Ala, and 3-AiB were good discriminators for CRC patients

a Symbol: “-” denotes studies where the specific information was not provided.

b Colorectal cancer.

c In samples corresponding to colorectal cancer, abbreviations are as follows. Ala: alanine; 2-AB: 2-aminobutyrate; 3-AiB: 3-aminoisobutyrate; ArA: arachidonic acid; Acet: acetate; Asp: aspartate; But: butyrate; Chol: choline; Cholt: cholesterol; Cit: citrate; Cr: creatine; Cys: cysteine; Fum: fumarate; Glc: glucose; Gln: glutamine; Glu: glutamate; Gly: glycine; Hip: hippurate; Isogln: isoglutamine; Kyn: kynurenate; Lac: lactate; Mal: malate; MI: myo-inositol; Myr: myristate; *p*-cre: *p*-cresol; PEG: polyethylene glycol; PC: phosphocholine; PE: phosphoethanolamine; Phe: phenylalanine; Pro: proline; Put: putrescine; SCFA: short-chain fatty acids; Tau: taurine; TCA: tricarboxylic acids; Tyr: tyrosine; Urd: uridine.

In the first group using fecal samples, two of these articles need to be highlighted: Bezabeh et al.^102^ and Monleón et al.,^103^ who investigated metabolic differences between feces samples from healthy controls and colorectal cancer patients. Both used NMR operating at different frequencies, and while Bezabeh et al.^102^ only found spectral differences between groups (using a 400 MHz spectrometer), Monleón et al.^103^ were able to find some biomarkers associated with colorectal cancer (using a 600 MHz spectrometer), such as acetate or butyrate. For NMR analyses, feces samples are not difficult to prepare,^102−104^ and therefore, it could be a simple way to study this disease in a less invasive way.

The second group related to tissue samples includes the works of Chan et al.,^105^ Piotto et al.,^106^ and Jiménez et al.^107^ (Table 2), who applied high resolution magic angle spinning (HR-MAS) NMR spectroscopy to study samples coming from tumors or adjacent normal mucosae obtained through biopsies. An advantage of using HR-MAS is that a smaller amount of sample is needed for the analysis and that the measurements are carried out directly in the solid state. However, handling tissue samples implies the use of an invasive collection method that contradicts one of the main advantages of applying NMR in metabolomics studies. HR-MAS analysis in tissue samples was able to find principally lactate and glucose, among others, as biomarkers for the disease.^105−107^ As previously discussed, some of these same biomarkers were also elucidated in the serum of patients with CRC^7,91,97^ but with the advantage that a noninvasive collection method was applied.

The third group includes the studies of Wang et al.^108^ and Kim et al.^109^ with urine samples. They employed NMR operating at 400 and 500 MHz, respectively, to assess metabolic changes in urine samples and were able to find some specific biomarkers, such as taurine, alanine, and 3-aminoisobutyrate, as well as distinguish between early stages of colorectal cancer and esophageal cancer.^108,109^ There are some studies, such as the one of Vahabi et al.,^98^ where they were also able to distinguish between different stages of colorectal cancer employing serum samples.

From Table 2, it is deduced that there is some variability on NMR operation frequencies as a function of the matrix chosen, but in terms of unraveled biomarkers, there is a trend that agrees with the tendency observed in serum samples: the Warburg effect is emphasized once again due to the increase of lactate and the decrease in glucose detected in most matrices.

**Figure 5:**
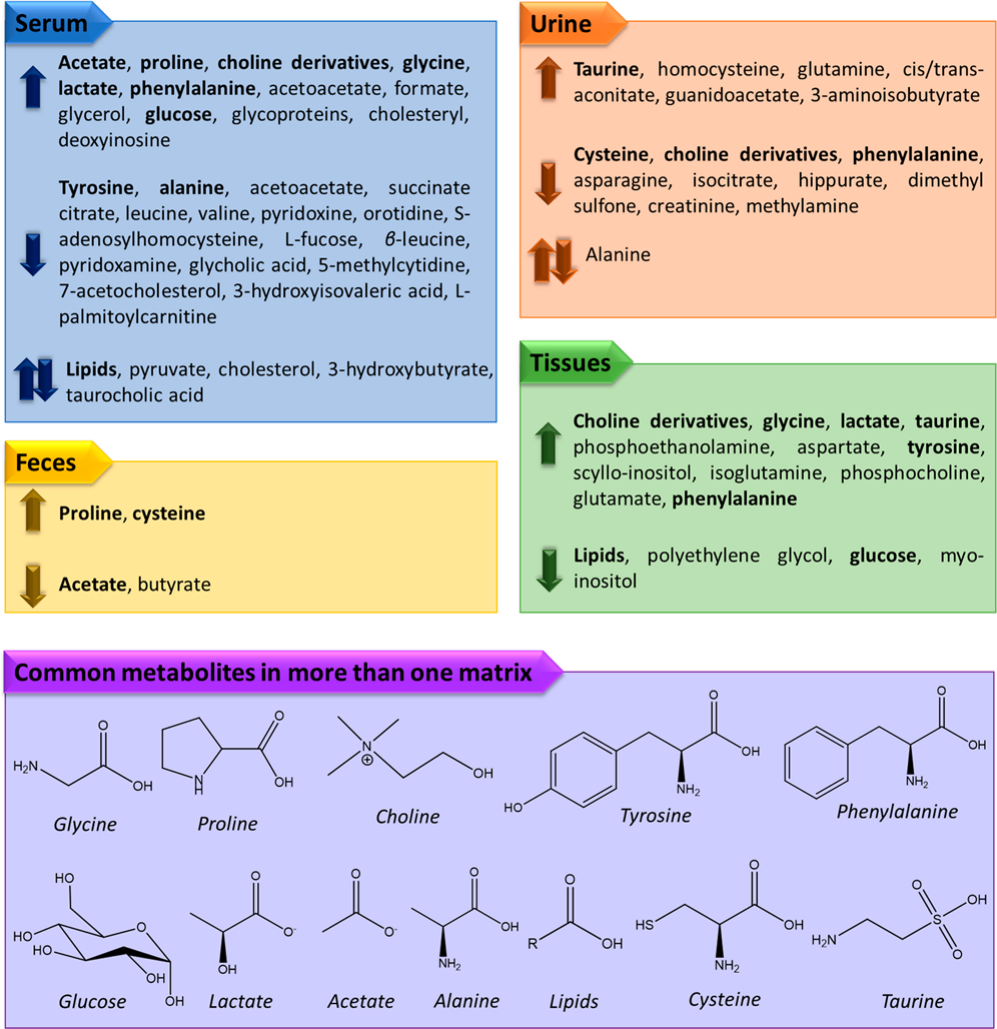
Biomarkers as a function of the matrix under study for colorectal cancer. Arrows pointing up or down reveal upregulated and downregulated biomarkers, respectively. Arrows pointing up and down simultaneously illustrate contradictory results on biomarkers that were reported to increase or decrease in different studies. Metabolites in bold have been detected in more than one matrix, and their molecular structures are given in the box below.

In Figure 5, we have summarized the different metabolites found as biomarkers as a function of the matrix under study. In addition, we have illustrated the different sets of metabolites with an arrow pointing upward or downward depending on whether the biomarker increases or decreases for the colorectal cancer group, respectively.

## Conclusions

NMR spectroscopy is presented as a powerful technique for the identification of specific metabolites even in complex mixtures, showing great applicability in the field of clinical metabolomics. There are currently scarce contributions of NMR metabolomics approaches in the study of colorectal cancer serum samples although, in general, all of them show promising outcomes. On the basis of the multiple statistical methods employed by each study, it can be concluded that there is no standard procedure among them for the identification of relevant biomarkers, which can lead to multiple conclusions, since data processing and data preparation are crucial steps to achieve correct interpretation of the results. Nevertheless, most studies discussed in this Review agreed on the role of colorectal cancer metabolites involved in glycolysis, specifically referring to the Warburg effect, which is a characteristic of carcinogenic samples. Also, alterations in the amino acids content and in the metabolism of glycerolipids and fatty acids were reported in most of the studies.

It would be of great interest to continue exploring the associated serum metabolic profiles to different stages of the disease, consolidating sample sizes, aims, and interest groups, and to increase the low number of studies in NMR metabolomics addressing this topic. Also, researchers should take into account the presence of other variables, such as the patient’s age, the occurrence of other diseases, and the physical state of the individual, since some investigations with those aspects have also shown encouraging outcomes.^106,108^ Finally, research in this field should be stimulated and correctly driven to understand and predict basic biological issues associated with colorectal cancer.
